# Incidence of depression in patients with cardiovascular disease and type 2 diabetes: a nationwide cohort study

**DOI:** 10.1007/s00392-023-02311-3

**Published:** 2023-10-10

**Authors:** Bochra Zareini, Katrine Kold Sørensen, Paul Blanche, Alexander C. Falkentoft, Emil Fosbøl, Lars Køber, Christian Torp-Pedersen

**Affiliations:** 1 Departments of Clinical Investigation and Cardiology and Department of Cardiology, North Zealand University Hospital, Dyrehavevej 29, 2400 Hillerød, Denmark; 2 Section of Biostatistics, Department of Public Health, University of Copenhagen, Copenhagen, Denmark; 3 Department of Cardiology, Zealand University Hospital, University of Copenhagen, Roskilde, Denmark; 4 Department of Cardiology, Rigshospitalet, University of Copenhagen, Copenhagen, Denmark

**Keywords:** Type 2 diabetes, Cardiovascular disease, Myocardial infarction, Depression, Stroke

## Abstract

**Background:**

Estimating how type 2 diabetes (T2D) affects the rate of depression in cardiovascular disease (CVD) can help identify high-risk patients. The aim is to investigate how T2D affects the rate of depression according to specific subtypes of CVD.

**Methods:**

Incident CVD patients, free of psychiatric disease, with and without T2D, were included from nationwide registries between 2010 and 2020. We followed patients from CVD diagnosis until the first occurrence of depression, emigration, death, 5 years, or end of study (December 31, 2021). We used time-dependent Poisson regression to estimate the incidence rates and rate ratios (IRR) of depression following subtypes of CVD with and without T2D. The model included age, sex, comorbidities, calendar year, T2D duration, educational level, and living situation as covariates.

**Results:**

A total of 165,096 patients were included; 45,845 had a myocardial infarction (MI), 63,691 had a stroke, 19,959 had peripheral artery disease (PAD), 35,568 had heart failure (HF), and 979 were diagnosed with 2 or more CVD subtypes (= > 2 CVD’s). Baseline T2D in each CVD subtype ranged from 11 to 17%. The crude incidence rate of depression per 1000 person-years (95% confidence intervals) was: MI + T2D: 131.1 (109.6;155.6), MI: 82.1 (65.3;101.9), stroke + T2D: 287.4 (255.1;322.6), stroke: 222.4(194.1;253.6), PAD + T2D: 173.6 (148.7;201.4), PAD:137.5 (115.5;162.5), HF + T2D: 244.3 (214.6;276.9), HF: 199.2 (172.5;228.9), =  > 2 CVD’s + T2D: 427.7 (388.1;470.2), =  > 2 CVD’s: 372.1 (335.2;411.9). The adjusted IRR of depression in MI, stroke, PAD, HF, and =  > 2 CVD’s with T2D compared to those free of T2D was: 1.29 (1.23;1.35), 1.09 (1.06;1.12), 1.18 (1.13;1.24), 1.05 (1.02;1.09), and 1.04 (0.85;1.27) (*p*-value for interaction < 0.001).

**Conclusion:**

The presence of T2D increased the rate of depression differently among CVD subtypes, most notable in patients with MI and PAD.

**Supplementary Information:**

The online version contains supplementary material available at 10.1007/s00392-023-02311-3.

## Introduction

The presence of depression in patients with type 2 diabetes (T2D) can directly or indirectly affect self-care, compliance, self-perceived health, quality of life, risk of hyperglycemia, development of incident macrovascular and microvascular complications, and risk of death [1]. Insight into the relationship between T2D and depression is essential to create appropriate awareness in physicians treating these patients. There is an increased risk of depression among patients with T2D. Cardiovascular disease (CVD) is the primary long-term risk for patients with T2D, and in this condition, the occurrence of depression is high [2, 3]. Several randomized controlled trials have investigated the effect of antidepressants or cognitive behavior therapy in patients with coronary artery disease, acute coronary syndrome, or acute myocardial infarction (MI) on reducing depression, recurrent MI, and mortality. The number of participants enrolled ranged from 101 to 2481, T2D patients comprised 30% of the population, and follow-up ranged from three months to 4 years [3–7]. The trials have shown that the rate is elevated in patients with MI and T2D compared to patients with MI free of T2D, but they did not include different subtypes of CVD [8, 9]. One meta-analysis based on two observational studies, including 90,412 and 144,216 patients, concluded that CVD was associated with an increased rate of depression but with no significant differences between patients developing stroke, peripheral artery disease (PAD) or chronic heart disease with or without T2D [10]. Also, heart failure (HF) was not investigated. There is no simple biological model that can explain the relationship between depression and cardiovascular disease. It can be attributed to a complex interplay of biological mechanisms, behavioral mechanisms, psychological coping mechanisms, and demographic as well as psychosocial factors [8]. Therefore, it is difficult to assume that the incidence of depression will be similar across different subtypes of CVD. Depression also affects compliance of preventive medical treatment, making patients vulnerable for CVD disease progression. While the primary focus for treatment of patients with T2D is on glycemic treatment and prevention of CVD, it is crucial to have awareness of depression. Detecting vulnerable CVD groups at risk is essential because effective management of depression in T2D patients has been shown to improve quality of life and glycemic control [11, 12]. Using the nationwide Danish registries, we aimed to: (1) Estimate the rate of depression in patients with T2D and MI, stroke, PAD, HF and patients who develop a combination or two or more CVD subtypes compared to those with the respective CVD subtype without T2D; (2) Determine if differences in age and sex drove the rate of depression; (3) Investigate how time since CVD diagnosis affects the rate of depression; (4) To compare the 5-year risk of all-cause death following a depression diagnosis between those with T2D and MI, stroke, PAD, HF, and a combination of two or more CVD subtypes and those without T2D.

## Methods

### Data sources

In Denmark, an identification number given at birth or immigration allows cross-linkage of health and administrative databases at the individual level and enables complete follow-up. Every citizen is granted equal access to the health care system, including primary and hospital care. Data for this study were cross-linked using six nationwide registries: (1) The Danish National Patient Registry contains information on all hospital admissions and outpatient contacts from 1977 onwards. According to The International Classification of Disease Ninth and Tenth Revision, each contact is coded with a primary diagnosis and one or more secondary diagnoses. Surgical procedures are coded using the Nordic Medico-Statistical Committee Classification of Surgical Procedures. (2) The Danish Psychiatric Central Research Register included data on psychiatric diagnoses for all inpatients and outpatients since 1995. (3) The Danish National Prescription Registry holds information (dosage, dates, and Anatomical Therapeutic Chemical [ATC] codes) on all prescriptions dispensed from a pharmacy since 1995. (4) The Danish Cause of Death Registry entails information on the date, cause, and place of death from 1970 onwards. (5) The Danish Population Registry contains information on the date of birth and sex. (6) Information on the living situation and educational level was extracted from the Civil Registration System and the Educational register.

### Study population

We included incident CVD patients (between the ages of 18 and 100) with and without T2D diagnosed between 2010 and 2020. To allow for a minimum of one-year follow-up, we stopped the inclusion at 31.12.2020. The subtypes of CVD were identified from in and outpatient contacts (including primary and secondary diagnoses) in the Danish National Patient Registry using date of discharge as the diagnosis date. CVD was defined as a first-time diagnosis of MI, stroke (including ischemic and hemorrhagic stroke), PAD, or HF. We included procedural codes for lower extremity revascularization procedures in defining the PAD diagnosis. Patients with a first-time diagnosis of HF and MI within the same hospital contact were regarded as HF patients. Patients receiving multiple first-time diagnoses during the same hospital contact compromised their own group and will be referred to the combination of two or more CVD subtypes throughout the manuscript. The specific diagnoses and the procedural codes used are listed in Supplementary Table S1.

### Definition of type 2 diabetes

We defined T2D as having a redeemed prescription of a glucose-lowering drug (ATC: A10) before inclusion. We excluded patients with type 1 diabetes, gestational diabetes, and polycystic ovarian syndrome based on the prescription patterns and age criteria. Patients were regarded as having type 1 diabetes if they redeemed only insulin prescriptions (ATC: A10A) before the age of 30 years. Women in monotherapy with metformin before age 40 were regarded as possible polycystic ovarian syndrome. Women redeeming glucose-lowering drugs 42 weeks before and 30 days after giving birth were regarded as gestational diabetes and thus excluded. Patients redeeming a prescription of Saxenda (Liraglutide, ATC: A10BJ02) were also excluded, as this drug was not approved for treating T2D patients in the study period of interest [13].

### Outcome

The primary outcome was incident depression, defined by the first use of an antidepressant (ATC: N06A, N05BA) or first hospital contact (including in- and out-hospital contacts, primary, and secondary diagnoses) for depression. Diagnosis codes used are available in Supplementary Table S1.

### Follow-up

Patients were included at the time of CVD diagnosis and followed until depression, 5 years after CVD development, emigration, all-cause death, or the end of the study (December 31, 2021). To estimate the 5-year risk of all-cause death following depression, we created a cohort of persons diagnosed with depression and alive at the time of the depression diagnosis. Persons diagnosed with depression were followed from the date of depression diagnosis until death, emigration, or the end of the study (December 31, 2021).

### Comorbidities and educational level

Comorbidities were identified from prior in- and out-hospital contacts up to 10 years prior to the date of inclusion. Information on concomitant medical therapy was obtained from dispensed prescriptions, as listed in the Danish National Prescription Registry, and defined by at least one redeemed prescription six months before the inclusion date. Hypertension was defined as treatment with at least two classes of anti-hypertensive drugs six months before inclusion as done previously [14]. Educational level was defined as the highest level of completed education and classified according to the Internal Standard Classification of Education.

### Statistical analysis

Baseline characteristics were described by the use of proportions for categorical variables and means and standard deviations (SD) or medians and interquartile ranges (IQR) for continuous variables. A reverse Kaplan–Meier estimator estimated the median follow-up time [15]. Crude incidence rates (IR) were calculated per 1000 person-years along with crude incidence rate ratios (IRR), and the exact Poisson method was used to compute 95% confidence intervals. To investigate how T2D affects the association between CVD subtypes (MI, stroke, PAD, HF, and combination of two or more CVD subtypes) and depression, a time-dependent Poisson regression model was estimated with an interaction term between T2D and CVD subtypes to compare the adjusted IRR of depression among patients with MI, stroke, PAD, HF, and a combination of two or more CVD disease subtypes compared to same CVD group but free of T2D. The model included the following covariates: age groups (< 60y, 60–80y, > 80), gender, comorbidities (atrial fibrillation, cancer, chronic obstructive pulmonary disease, hypertension, chronic kidney disease, and liver disease), CVD duration (0–1y, > 1–3y, > 3–5y), a living situation known at the time of CVD diagnosis (living alone versus not living alone), and educational level known at the time of CVD diagnosis. Age, comorbidities, and CVD duration were included as time-updated covariates. All estimates are reported with 95% confidence intervals. To investigate potential differences across subgroups, we reproduced the abovementioned analysis in subgroups of specific patient characteristics to assess whether the IRRs of depression were similar across age groups (< 60y, 60–80y, > 80), gender, and duration of CVD (0–1y, > 1–3y, > 3–5y). To describe mortality after depression, we report the 5-year risk of all-cause mortality following a diagnosis of depression among CVD subtypes with and without T2D within the study period using the Kaplan–Meier estimator [16]. Patients developing more than one CVD subtype prior to diagnosis of depression compromised their own subgroup. All analyses were performed in R version 4.2.1 [17].

### Ethics

By law, registered-based research has been granted permission to be performed without ethical approval or informed consent. Therefore, the project has been approved by the Knowledge Centre on Data Protection Compliance—The Capitol Region of Denmark (approval number: P-2010-191).

## Results

### Study population

**Fig. 1:**
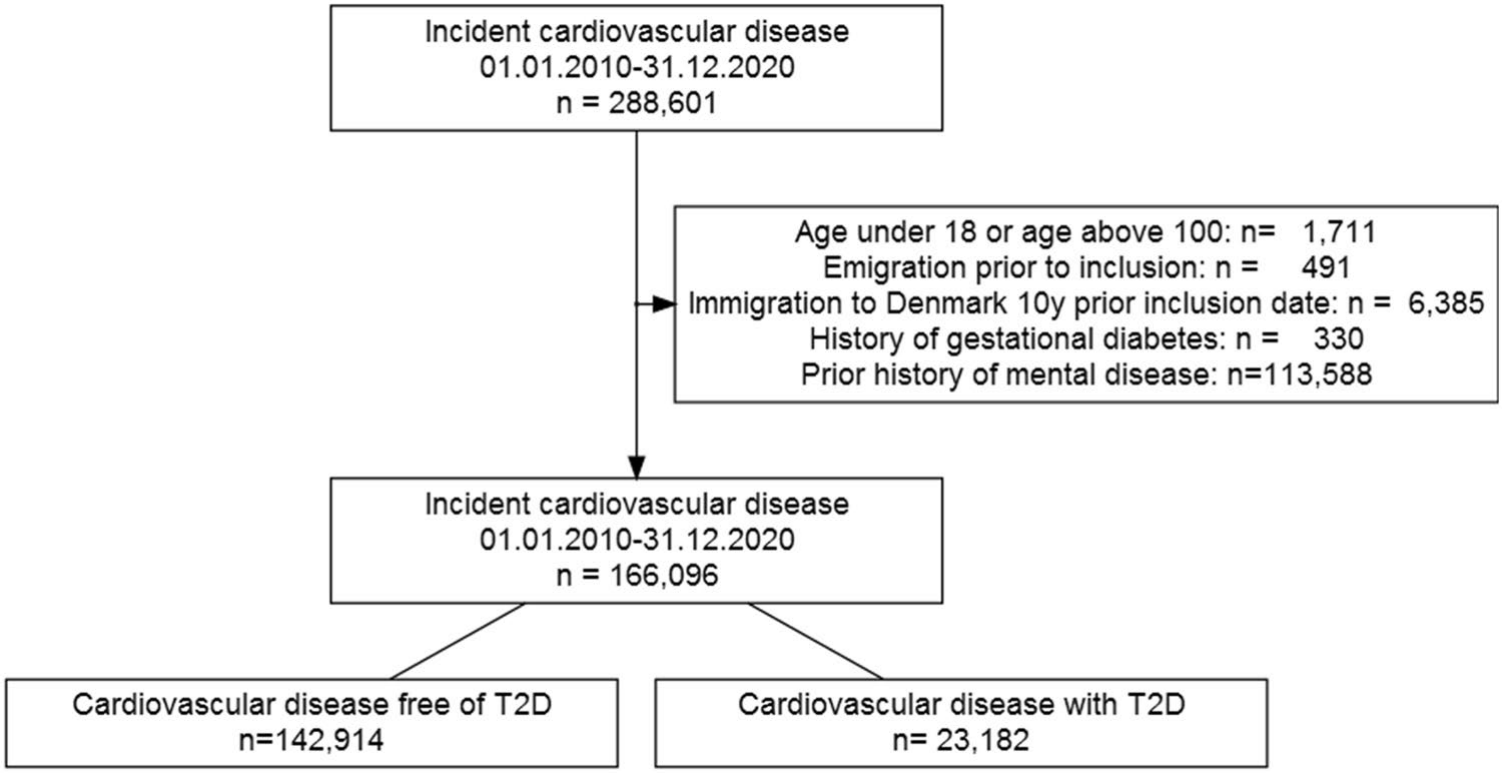
Flowchart of the study cohort. *n* number, *T2D* type 2 diabetes

The study population consisted of 166,096 patients. As shown in Fig. 1, 45,845 had MI, 63,691 had a stroke, 19,959 had PAD, 35,568 had HF, and 979 were diagnosed with a combination of two or more CVD subtypes. Baseline T2D within each group ranged from 11 to 17%. Baseline characteristics of the cohort according to the subtype of CVD with and without T2D are shown in Table 1. Patients with CVD and T2D compared to CVD patients free of T2D were characterized by being older, more likely to be male, living alone, less likely to have a master's or Ph.D., and more likely to receive concomitant medication.

**Table 1:** Patient characteristics of the study population at time of CVD diagnosis

	MI		Stroke		PAD		HF		= > 2 CVD’s	
	No T2D (*n* = 40,486)	T2D (*n* = 5359)	No T2D (*n* = 56,729)	T2D (*n* = 6962)	No T2D (*n* = 15,420)	T2D (*n* = 4593)	No T2D (*n* = 29,449)	T2D (*n* = 6119)	No T2D (*n* = 830)	T2D (*n* = 149)
Age (median (IQR))	66.9 (56.9, 75.9)	70.5 (61.8, 78.3)	71.8 (61.4, 80.7)	73.9 (66.1, 81.0)	72 (64.1, 79.6)	71.6 (64.3, 78.3)	76.7 (66.5, 85.1)	76 (68.4, 82.8)	77.4 (67.7, 85.0)	74.9 (68.6, 82.0)
T2D duration (years, median (IQR))		8 ( 3.7, 13.9)		8.5 ( 4.1, 14.1)		10.2 ( 4.6, 16.1)		8.2 ( 4, 14)		8.2 ( 4.5, 14.3)
Males	28,297 (69.9)	3766 (70.3)	31,682 (55.8)	4347 (62.4)	8539 (55.4)	3271 (71.2)	17,596 (59.8)	3915 (64.0)	495 (59.6)	94 (63.1)
Basic education	14,549 (35.9)	2396 (44.7)	22,605 (39.8)	3261 (46.8)	6808 (44.2)	2137 (46.5)	13,854 (47.0)	3186 (52.1)	447 (53.9)	82 (55.0)
General upper secondary education	17,514 (43.3)	2158 (40.3)	22,612 (39.9)	2706 (38.9)	6278 (40.7)	1897 (41.3)	10,763 (36.5)	2189 (35.8)	250 (30.1)	52 (34.9)
Bachelor level education	6336 (15.6)	617 (11.5)	8556 (15.1)	767 (11.0)	1844 (12.0)	460 (10.0)	3533 (12.0)	579 (9.5)	95 (11.4)	10 (6.7)
Masters or PhD	2087 (5.2)	188 (3.5)	2956 (5.2)	228 (3.3)	490 (3.2)	99 (2.2)	1299 (4.4)	165 (2.7)	38 (4.6)	5 (3.4)
Living alone	8457 (20.9)	1178 (22.0)	14,719 (25.9)	1860 (26.7)	4172 (27.1)	1180 (25.7)	9219 (31.3)	1761 (28.8)	318 (38.3)	54 (36.2)
Hypertension	14,051 (34.7)	3698 (69.0)	21,378 (37.7)	5072 (72.9)	7826 (50.8)	3587 (78.1)	15,595 (53.0)	5016 (82.0)	450 (54.2)	119 (79.9)
Atrial fibrillation	2598 (6.4)	582 (10.9)	5663 (10.0)	1056 (15.2)	1662 (10.8)	631 (13.7)	8708 (29.6)	2020 (33.0)	156 (18.8)	30 (20.1)
Cancer	5327 (13.2)	885 (16.5)	9609 (16.9)	1378 (19.8)	3032 (19.7)	807 (17.6)	6359 (21.6)	1381 (22.6)	141 (17.0)	32 (21.5)
COPD	10,116 (25.0)	1629 (30.4)	13,114 (23.1)	1880 (27.0)	4853 (31.5)	1295 (28.2)	10,443 (35.5)	2562 (41.9)	238 (28.7)	60 (40.3)
CKD	1091 (2.7)	583 (10.9)	1453 (2.6)	769 (11.0)	821 (5.3)	751 (16.4)	1680 (5.7)	1035 (16.9)	44 (5.3)	25 (16.8)
Liver disease	93 (0.2)	43 (0.8)	242 (0.4)	97 (1.4)	99 (0.6)	53 (1.2)	173 (0.6)	86 (1.4)	0 (0.0)	0 (0.0)
ASA	6437 (15.9)	2008 (37.5)	7751 (13.7)	2095 (30.1)	7119 (46.2)	2267 (49.4)	6540 (22.2)	2328 (38.0)	229 (27.6)	55 (36.9)
Statins	7680 (19.0)	3127 (58.4)	9148 (16.1)	3715 (53.4)	7895 (51.2)	3110 (67.7)	6348 (21.6)	3666 (59.9)	211 (25.4)	73 (49.0)
ACE/ARB	10,887 (26.9)	3410 (63.6)	15,438 (27.2)	4346 (62.4)	6262 (40.6)	3246 (70.7)	11,849 (40.2)	4078 (66.6)	316 (38.1)	95 (63.8)
Beta-blockers	6164 (15.2)	1521 (28.4)	9165 (16.2)	2037 (29.3)	3367 (21.8)	1489 (32.4)	10,721 (36.4)	2851 (46.6)	242 (29.2)	67 (45.0)
Ca channel blockers	6975 (17.2)	1847 (34.5)	9649 (17.0)	2318 (33.3)	4333 (28.1)	1871 (40.7)	5817 (19.8)	2169 (35.4)	183 (22.0)	51 (34.2)
Thiazide	4158 (10.3)	796 (14.9)	6283 (11.1)	1094 (15.7)	2410 (15.6)	798 (17.4)	4133 (14.0)	990 (16.2)	119 (14.3)	26 (17.4)
MRA	473 (1.2)	183 (3.4)	772 (1.4)	276 (4.0)	494 (3.2)	293 (6.4)	2534 (8.6)	763 (12.5)	29 (3.5)	20 (13.4)
Digoxin	475 (1.2)	180 (3.4)	1352 (2.4)	362 (5.2)	463 (3.0)	190 (4.1)	2720 (9.2)	716 (11.7)	52 (6.3)	17 (11.4)
Loop diuretics	2107 (5.2)	871 (16.3)	3644 (6.4)	1128 (16.2)	1936 (12.6)	1115 (24.3)	9894 (33.6)	3020 (49.4)	177 (21.3)	56 (37.6)
ADPi	7054 (17.4)	2150 (40.1)	10,023 (17.7)	2490 (35.8)	7811 (50.7)	2479 (54.0)	7192 (24.4)	2514 (41.1)	270 (32.5)	65 (43.6)
Vitamin K antagonist	1133 (2.8)	273 (5.1)	2467 (4.3)	523 (7.5)	900 (5.8)	360 (7.8)	4628 (15.7)	1120 (18.3)	72 (8.7)	22 (14.8)
NOAC's	564 (1.4)	146 (2.7)	1360 (2.4)	258 (3.7)	477 (3.1)	169 (3.7)	2481 (8.4)	632 (10.3)	29 (3.5)	6 (4.0)
Insulin	0 (0.0)	1372 (25.6)	0 (0.0)	1760 (25.3)	0 (0.0)	1723 (37.5)	0 (0.0)	1580 (25.8)	0 (0.0)	38 (25.5)
Metformin	0 (0.0)	3573 (66.7)	0 (0.0)	4496 (64.6)	0 (0.0)	2844 (61.9)	0 (0.0)	3989 (65.2)	0 (0.0)	105 (70.5)
Sulfonylurea	0 (0.0)	956 (17.8)	0 (0.0)	1166 (16.7)	0 (0.0)	775 (16.9)	0 (0.0)	1109 (18.1)	0 (0.0)	41 (27.5)
DDP-4 inhibitors	0 (0.0)	655 (12.2)	0 (0.0)	827 (11.9)	0 (0.0)	592 (12.9)	0 (0.0)	774 (12.6)	0 (0.0)	21 (14.1)
GLP1-RA/SGLT2i	0 (0.0)	558 (10.4)	0 (0.0)	597 (8.6)	0 (0.0)	525 (11.4)	0 (0.0)	516 (8.4)	0 (0.0)	14 (9.4)

*n* number, *IQR* interquartile range, *MI* myocardial infarction, *PAD* peripheral artery disease, *HF* heart failure, *=  > 2 CVD’s* a combination of two or more CVD subtypes, *CVD* cardiovascular disease, *COPD* chronic obstructive pulmonary disease, *CKD* chronic kidney disease, *ACE* angiotensin-converting enzyme, *ARB* angiotensin II receptor blocker, *ADP* adenine diphosphate receptor, *NOAC’s* new oral anticoagulants, *MRA* mineralocorticoid receptor antagonists, *DPP-4* dipeptidyl peptidase-4, *GLP-1* glucagon-like peptide-1, *SGLT2* sodium–glucose-cotransporter-2

### Type 2 diabetes and the rate of depression

**Fig. 2:**
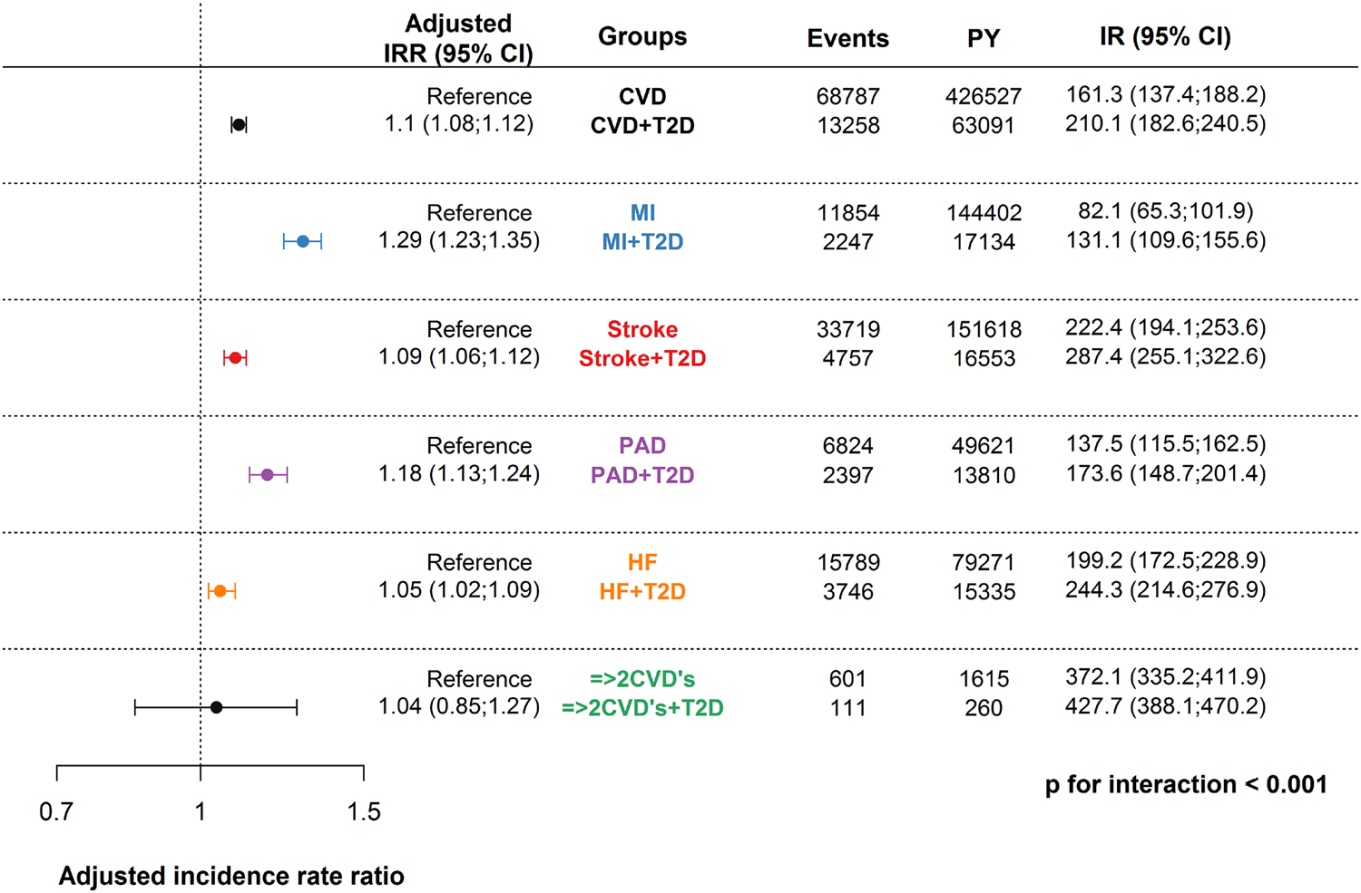
Crude incidence rates and adjusted incidence rate ratios of depression among subtypes of cardiovascular disease with type 2 diabetes compared to subtypes of cardiovascular disease free of type 2 diabetes. The incidence rate ratios were adjusted for age, gender, educational level, living situation, and comorbidities (atrial fibrillation, cancer, chronic obstructive pulmonary disease, hypertension, chronic kidney disease and liver disease). *T2D* type 2 diabetes, *CVD* cardiovascular disease, *PY* person-years, *IR* incidence rate per 1000 person-years, *CI* confidence interval, *MI* myocardial infarction, *HF* heart failure, *PAD* peripheral artery disease, *=  > 2 CVD’s* a combination of two or more cardiovascular disease subtypes

The median follow-up time was: 4.4 years (IQR: 1.9; 5.0), in which 22,103 (13%) experienced the outcome of depression (Fig. 2). Only 1,082 patients were first diagnosed with an in-/out-hospital contact, and a redeemed prescription of an antidepressant was first identified in 18,883 patients. The events, the crude incidence rates, and adjusted IRRs of depression are shown in Fig. 2. T2D increased the incidence rate ratio of depression among all CVD subtypes. There were differences in crude incidence rates among the CVD subtypes, where patients with multiple CVD subtypes had a much higher incidence of depression overall compared to the other CVD subtypes. Effects among age groups and gender are shown in Table S2, and Figure S1 in the supplementary material. T2D increased the rate of depression in all age groups, but the effect of T2D diminished with increasing age. We did not observe significant differences between males and females in the other CVD subtypes when considering age. It should be noted that the adjusted incidence rate ratios were generally lower than the unadjusted ones, suggesting that confounding factors may explain some of the observed associations.

### Duration of cardiovascular disease and development of depression

**Fig. 3:**
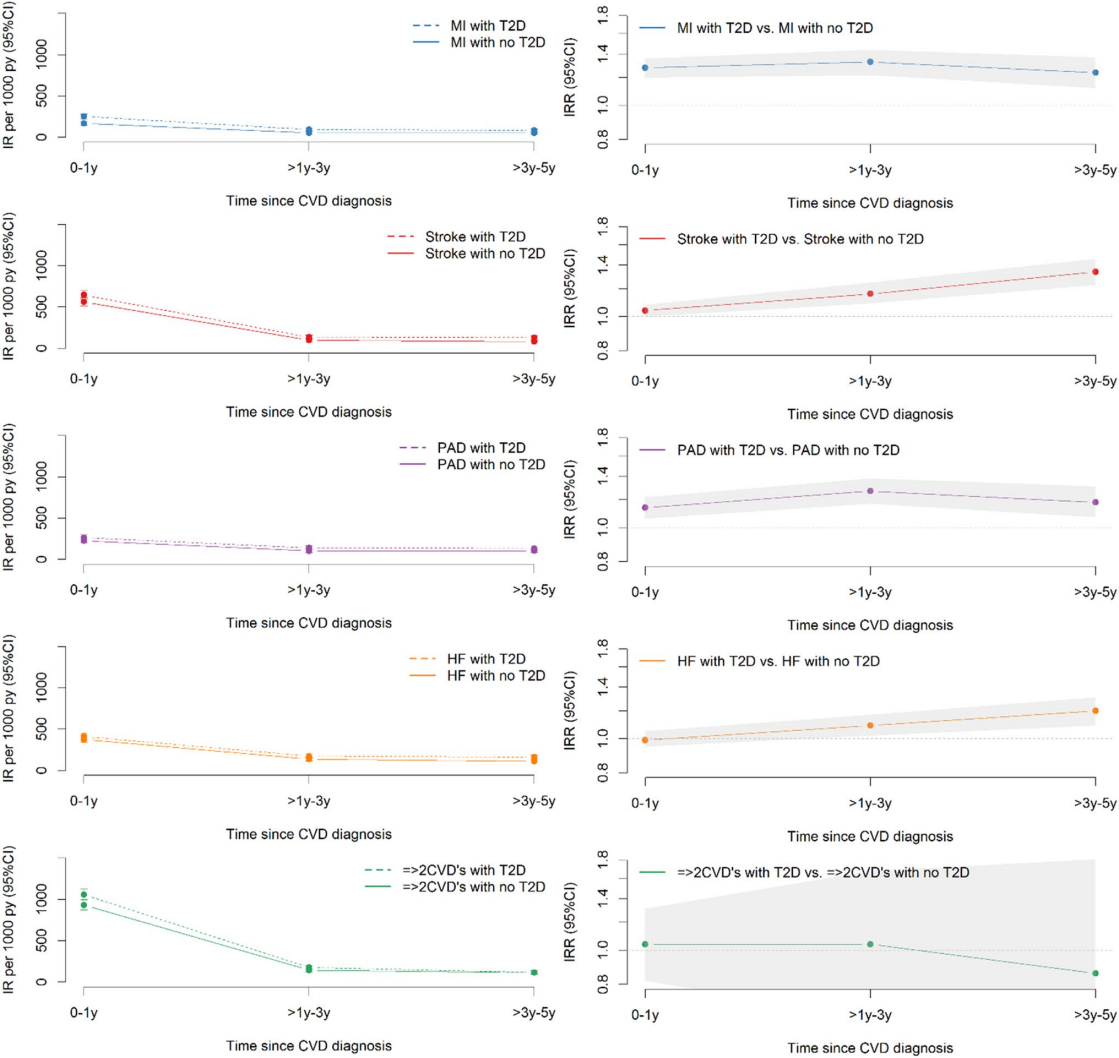
Crude incidence rates and adjusted incidence rate ratios of depression among subtypes of cardiovascular disease with type 2 diabetes compared to subtypes of cardiovascular disease free of type 2 diabetes according to duration of cardiovascular disease. The incidence rate ratios were adjusted for age, gender, educational level, living situation, and comorbidities (atrial fibrillation, cancer, chronic obstructive pulmonary disease, hypertension, chronic kidney disease and liver disease). *T2D* type 2 diabetes, *CVD* cardiovascular disease, *PY* person-years, *IR* incidence rate per 1000 person-years, *CI* confidence interval, *MI* myocardial infarction, *HF* heart failure, *PAD* peripheral artery disease, *=  > 2 CVD’s* a combination of two or more cardiovascular disease subtypes

The effect of T2D remained constant and elevated across all 5 years after the development of MI, stroke, PAD, or HF. The adjusted IRRs of depression 0–1 year, > 1–3 years, and > 3–5 years after CVD diagnosis are shown in Fig. 3 and Table S4 in the supplementary material. The absolute rates were the highest in the 0–1 year after CVD diagnosis and dropped in the rest of the follow-up period, but the effect of T2D on depression remained elevated with time. For patients with stroke and HF, the effect of T2D on depression slightly increased over time.

### Risk of all-cause death following a diagnosis of depression

**Fig. 4:**
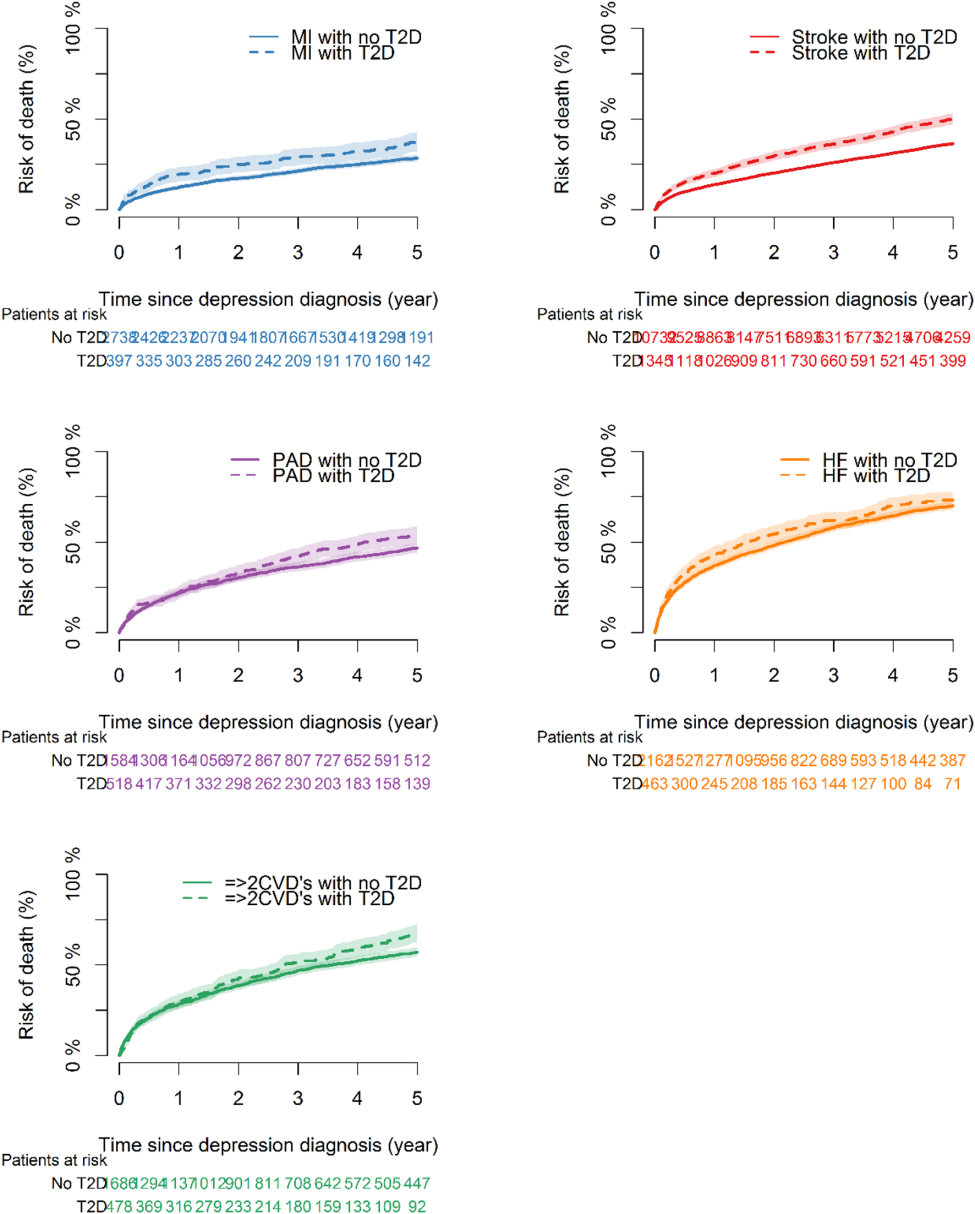
Risk of all-cause death following diagnosis of depression in patients with cardiovascular disease with and without type 2 diabetes. *T2D* type 2 diabetes, *MI* myocardial infarction, *HF* heart failure, *PAD* peripheral artery disease, *=  > 2 CVD’s* a combination of two or more cardiovascular disease subtypes

A total of 22,103 patients with depression were included in the second cohort. Demographic characteristics and comorbidities at the time of inclusion (time of depression diagnosis) are depicted in Table S6 in the supplementary material. Patients with T2D were older, more likely to be male, and suffer from comorbidities. Following a diagnosis of depression, the median survival time was 4.0 years (IQR: 3.2; 6.3), and 10,196 patients died during the study period. The risk of all-cause death following depression in groups of CVD subtypes is depicted in Fig. 4. The 5-year risk of all-cause death for patients with MI, stroke, PAD, HF, or a combination of two or more CVD subtypes, and T2D was: 43.3% (38.6:47.9), 51.1% (48.1;54.0), 57.3% (52.9;61.7), 73.0% (69.0;77.0), and 61.1% (39.5;82.8), respectively. The 5-year risk of all-cause death for patients with MI, stroke, PAD, HF, or a combination of two or more CVD subtypes free of T2D was: 32.2% (30.4;33.9), 37.1% (36.1;38.1), 48.4% (45.9;51.0), 68.8% (66.8;70.8), and 58.5% (49.4;67.6), respectively. T2D among patients with CVD significantly increased the 5-year risk of all-cause death following a diagnosis of depression.

## Discussion

Depression was common in patients with T2D and CVD, and T2D was associated with a higher rate of depression in all subtypes of CVD compared to CVD patients free of T2D. Furthermore, the association remained present with longer CVD duration. Once depression was diagnosed, T2D increased the 5-year risk of all-cause death, with the highest risk observed among HF patients.

One meta-analysis, based on two cohort studies, was conducted to investigate the impact of depression following CVD in patients with T2D [10]. They found a significantly increased rate of depression following CVD (coronary heart disease, cerebrovascular disease, myocardial infarction, or peripheral artery disease), with no differences between the different types of CVD. The first study included in the meta-analysis, by Jacob and Kostev, used patient records from general practitioners, and used diagnosis codes to define T2D and the outcome of depression, but found no significant difference in the rate of depression between the development of the subtype of CVD (coronary heart disease and of stroke) [18]. The second study, by Pan et al., included lower limb amputation in the definition of cardiovascular disease and found that the rates of depression were slightly enhanced by cardiovascular disease. However, they omitted HF and only distinguished between stroke and CVD [19]. Both studies defined the outcome of depression by in-hospital and out-hospital contacts. Only 2% of the outcome in our cohort received a depression diagnosis with an in-hospital or out-hospital contact. Our definition increased the sensitivity of the outcome, enabling a high accuracy of the effect estimate and testing for a possible hierarchal order among the rates of depression following CVD. T2D increased the rate of depression similarly across all subtypes of CVD. However, the magnitude of crude incidence rates of depression among the CVD subtypes free of T2D was different among the CVD subtypes. The difference in incidence rates of depression among patients with MI, HF, stroke, and PAD could be explained by differences in underlying mechanism, differences in behavioral risk factors of depression, differences in the invalidating capacity, or differences in the provider's ability to identify depression among these groups of patients. Since the effect of T2D was similar across all CVD subtypes, one might speculate that the level of cerebral microvascular dysfunction is independent of the CVD subtype.

Macrovascular and microvascular dysfunctions are critical consequences of hyperglycemia and insulin resistance [20–23]. This dysfunction is associated with an increased risk of cardiovascular diseases, including HF [24, 25]. Microvascular dysfunction has been associated with HF, nephropathy, and peripheral neuropathy, but especially cerebral microvascular dysfunction is associated with depression and dementia [24, 26–29].

The unified drop-in crude depression rates 12 months after the development of CVD could be a marker of the temporal incidence of depression or perhaps how depression is detected and managed in Denmark. National guidelines all recommend that patients following a diagnosis of MI, HF, and stroke receive a psychological evaluation following diagnosis [8]. No guidelines exist for patients with PAD or following lower limb amputations in Denmark. However, the adjusted IRR of depression in patients with PAD was as large as the IRR in patients with MI, and PAD could potentially be underdiagnosed due to differences in screening practice, or less severe PAD is not associated with the development of depression in a similar way as the other types of CVD. The difference could also result from providers' ability to detect or ask about depression. For example, providers could be prone to ask stroke patients about depressive symptoms and not recognize them in HF due to the shared symptoms (fatigue, loss of appetite, weight gain) [30]. The risk of death following depression diagnosis was the highest among HF patients, which has been investigated extensively before, including HF patients without T2D [31–34]. The strengths of this study are the nationwide completeness of data and the number of patients included. In addition, we used well-validated measures to asses exposure (T2D and diagnosis of CVD) and outcome (depression) [35–38]. However, since we relied on the prescription of antidiabetic drugs, antidepressants, and benzodiazepines, all patients with diet-treated T2D or mild depression were excluded, limiting the study's external validity. Additionally, we have reduced the sensitivity of depression diagnosis when including anxiety disorders in the definition as these conditions also are treated with antidepressants. Finally, we have presented an association, not causation, and residual confounding cannot be ruled out since we lacked critical information on behavioral risk factors, such as smoking, body mass index, and exercise. The severity of the CVD could also not be assessed, especially the etiology, severity, and symptoms of HF, stroke, and PAD.

## Conclusion

Depression was common in patients with T2D and CVD, and T2D was associated with a higher rate of depression in all subtypes of CVD compared to CVD patients free of T2D, but significantly higher among patients with MI and PAD. Furthermore, the association remained present with longer CVD duration. Once depression was diagnosed, T2D increased the 5-year risk of all-cause death in addition to all CVD subtypes, with the highest risk in HF patients.

## Supplementary Information

Below is the link to the electronic supplementary material.

Supplementary file1 (DOCX 492 KB)

## Data Availability

The data-sets generated during and/or analyzed in the current study are not available due to the data protection policies issued by Statistics Denmark.

## Abbreviations

T2D: Type 2 diabetes
CVD: Cardiovascular disease
MI: Myocardial infarction
HF: Heart failure
PAD: Peripheral artery disease
IQR: Interquartile range

## References

[1] Bădescu S, Tătaru C, Kobylinska L, Georgescu E, Zahiu D, Zăgrean A, Zăgrean L (2016) The association between diabetes mellitus and depression. J Med Life 9:120–125 27453739 PMC4863499

[2] Mezuk B, Eaton WW, Albrecht S, Golden SH (2008) Depression and type 2 diabetes over the lifespan: a meta-analysis. Diabetes Care 31:2383–2390 19033418 10.2337/dc08-0985 PMC2584200

[3] Glassman AH, O’Connor CM, Califf RM, Swedberg K, Schwartz P, Bigger JT, Krishnan KRR, van Zyl LT, Swenson JR, Finkel MS, Landau C, Shapiro PA, Pepine CJ, Mardekian J, Harrison WM, Barton D, Mclvor M, Sertraline Antidepressant Heart Attack Randomized Trial (SADHEART) Group (2002) Sertraline treatment of major depression in patients with acute MI or unstable angina. JAMA 288:701–709 12169073 10.1001/jama.288.6.701

[4] Writing Committee for the ENRICHD Investigators (2003) Effects of treating depression and low perceived social support on clinical events after myocardial infarction the enhancing recovery in coronary heart disease patients (ENRICHD) randomized trial. JAMA 289:3106–3116 12813116 10.1001/jama.289.23.3106

[5] Lespérance F, Frasure-Smith N, Koszycki D, Laliberté M-A, van Zyl LT, Baker B, Swenson JR, Ghatavi K, Abramson BL, Dorian P, Guertin M-C, Guertin M-C, CREATE Investigators for the (2007) Effects of citalopram and interpersonal psychotherapy on depression in patients with coronary artery disease: the Canadian cardiac randomized evaluation of antidepressant and psychotherapy efficacy (CREATE) trial. JAMA 297:367 17244833 10.1001/jama.297.4.367

[6] Blumenthal JA, Sherwood A, Babyak MA, Watkins LL, Smith PJ, Hoffman BM, O’Hayer CVF, Mabe S, Johnson J, Doraiswamy PM, Jiang W, Schocken DD, Hinderliter AL (2012) Exercise and pharmacological treatment of depressive symptoms in patients with coronary heart disease. J Am Coll Cardiol 60:1053–1063 22858387 10.1016/j.jacc.2012.04.040 PMC3498445

[7] O’Connor CM, Jiang W, Kuchibhatla M, Silva SG, Cuffe MS, Callwood DD, Zakhary B, Stough WG, Arias RM, Rivelli SK, Krishnan R (2010) Safety and efficacy of sertraline for depression in patients with heart failure. J Am Coll Cardiol 56:692–699 20723799 10.1016/j.jacc.2010.03.068 PMC3663330

[8] Hare DL, Toukhsati SR, Johansson P, Jaarsma T (2014) Depression and cardiovascular disease: a clinical review. Eur Heart J 35:1365–1372 24282187 10.1093/eurheartj/eht462

[9] Wynn A (1967) unwarranted emotional distress in men with ischaemic heart disease (IHD). Med J Aust 2:847–851 6081071 10.5694/j.1326-5377.1967.tb74314.x

[10] Nouwen A, Adriaanse MC, van Dam K, Iversen MM, Viechtbauer W, Peyrot M, Caramlau I, Kokoszka A, Kanc K, de Groot M, Nefs G, Pouwer F (2019) Longitudinal associations between depression and diabetes complications: a systematic review and meta-analysis. Diabet Med 36:1562–1572 31215077 10.1111/dme.14054

[11] Semenkovich K, Brown ME, Svrakic DM, Lustman PJ (2015) Depression in type 2 diabetes mellitus: prevalence, impact, and treatment. Drugs 75:577–587 25851098 10.1007/s40265-015-0347-4

[12] Brieler JA, Lustman PJ, Scherrer JF, Salas J, Schneider FD (2016) Antidepressant medication use and glycaemic control in co-morbid type 2 diabetes and depression. FAMPRJ 33:30–36 10.1093/fampra/cmv100 26743722

[13] SAXENDA (liraglutide [rDNA origin] injection). 40

[14] Olesen JB, Lip GYH, Hansen ML, Hansen PR, Tolstrup JS, Lindhardsen J, Selmer C, Ahlehoff O, Olsen A-MS, Gislason GH, Torp-Pedersen C (2011) Validation of risk stratification schemes for predicting stroke and thromboembolism in patients with atrial fibrillation: nationwide cohort study. BMJ 342:124 10.1136/bmj.d124 PMC3031123 21282258

[15] Gillespie BW, Chen Q, Reichert H, Franzblau A, Hedgeman E, Lepkowski J, Adriaens P, Demond A, Luksemburg W, Garabrant DH (2010) Estimating population distributions when some data are below a limit of detection by using a reverse Kaplan–Meier estimator. Epidemiology 21:S64–S70 20386104 10.1097/EDE.0b013e3181ce9f08

[16] Uno H, Claggett B, Tian L, Inoue E, Gallo P, Miyata T, Schrag D, Takeuchi M, Uyama Y, Zhao L, Skali H, Solomon S, Jacobus S, Hughes M, Packer M, Wei L-J (2014) Moving beyond the hazard ratio in quantifying the between-group difference in survival analysis. J Clin Oncol 32:2380–2385 24982461 10.1200/JCO.2014.55.2208 PMC4105489

[17] R Core Team. R: A Language and Environment for Statistical Computing

[18] Jacob L, Kostev K (2016) Prevalence of depression in type 2 diabetes patients in German primary care practices. J Diabetes Complications 30:432–437 26790576 10.1016/j.jdiacomp.2015.12.013

[19] Pan H-H, Li C-Y, Chen P-C, Lee M-D, Liang C-Y, Hou W-H, Wang K-Y (2012) Contributions of diabetic macro-vascular complications and hip fracture to depression onset in elderly patients with diabetes: an 8-year population-based follow-up study. J Psychosom Res 73:180–184 22850257 10.1016/j.jpsychores.2012.06.003

[20] van Sloten TT, Henry RMA, Dekker JM, Nijpels G, Unger T, Schram MT, Stehouwer CDA (2014) Endothelial dysfunction plays a key role in increasing cardiovascular risk in type 2 diabetes: the hoorn study. Hypertension 64:1299–1305 25225211 10.1161/HYPERTENSIONAHA.114.04221

[21] Forouhi NG, Luan J, Hennings S, Wareham NJ (2007) Incidence of type 2 diabetes in England and its association with baseline impaired fasting glucose: the Ely study 1990–2000. Diabet Med 24:200–207 17257284 10.1111/j.1464-5491.2007.02068.x

[22] Schram MT, Henry RMA, van Dijk RAJM, Kostense PJ, Dekker JM, Nijpels G, Heine RJ, Bouter LM, Westerhof N, Stehouwer CDA (2004) Increased central artery stiffness in impaired glucose metabolism and type 2 diabetes: the hoorn study. Hypertension 43:176–181 14698999 10.1161/01.HYP.0000111829.46090.92

[23] Mostaza JM, Lahoz C, Salinero-Fort MA, de Burgos-Lunar C, Laguna F, Estirado E, García-Iglesias F, González-Alegre T, Cornejo-Del-Río V, Sabín C, López S (2015) Carotid atherosclerosis severity in relation to glycemic status: a cross-sectional population study. Atherosclerosis 242:377–382 26275375 10.1016/j.atherosclerosis.2015.07.028

[24] Lee JF, Barrett-O’Keefe Z, Garten RS, Nelson AD, Ryan JJ, Nativi JN, Richardson RS, Wray DW (2016) Evidence of microvascular dysfunction in heart failure with preserved ejection fraction. Heart 102:278–284 26567228 10.1136/heartjnl-2015-308403 PMC4866903

[25] Gamrat A, Surdacki MA, Chyrchel B, Surdacki A (2020) Endothelial dysfunction: a contributor to adverse cardiovascular remodeling and heart failure development in type 2 diabetes beyond accelerated atherogenesis. J Clin Med 9:2090 32635218 10.3390/jcm9072090 PMC7408687

[26] Buysschaert M, Medina JL, Bergman M, Shah A, Lonier J (2015) Prediabetes and associated disorders. Endocrine 48:371–393 25294012 10.1007/s12020-014-0436-2

[27] Wong M-S, Gu K, Heng D, Chew S-K, Chew L-S, Tai ES (2003) The Singapore impaired glucose tolerance follow-up study: does the ticking clock go backward as well as forward? Diabetes Care 26:3024–3030 14578234 10.2337/diacare.26.11.3024

[28] Ford ES, Zhao G, Li C (2010) Pre-diabetes and the risk for cardiovascular disease. J Am Coll Cardiol 55:1310–1317 20338491 10.1016/j.jacc.2009.10.060

[29] van Sloten TT, Sedaghat S, Carnethon MR, Launer LJ, Stehouwer CDA (2020) Cerebral microvascular complications of type 2 diabetes: stroke, cognitive dysfunction, and depression. Lancet Diabetes Endocrinol 8:325–336 32135131 10.1016/S2213-8587(19)30405-X PMC11044807

[30] Brouwers C, Christensen SB, Damen NL, Denollet J, Torp-Pedersen C, Gislason GH, Pedersen SS (2016) Antidepressant use and risk for mortality in 121,252 heart failure patients with or without a diagnosis of clinical depression. Int J Cardiol 203:867–873 26599753 10.1016/j.ijcard.2015.11.032

[31] Rutledge T, Reis VA, Linke SE, Greenberg BH, Mills PJ (2006) Depression in heart failure. J Am Coll Cardiol 48:1527–1537 17045884 10.1016/j.jacc.2006.06.055

[32] Fan H, Yu W, Zhang Q, Cao H, Li J, Wang J, Shao Y, Hu X (2014) Depression after heart failure and risk of cardiovascular and all-cause mortality: a meta-analysis. Prev Med 63:36–42 24632228 10.1016/j.ypmed.2014.03.007

[33] Sokoreli I, de Vries JJG, Pauws SC, Steyerberg EW (2016) Depression and anxiety as predictors of mortality among heart failure patients: systematic review and meta-analysis. Heart Fail Rev 21:49–63 26572543 10.1007/s10741-015-9517-4

[34] Adelborg K, Schmidt M, Sundbøll J, Pedersen L, Videbech P, Bøtker HE, Egstrup K, Sørensen HT (2016) Mortality risk among heart failure patients with depression: a nationwide population-based cohort study. J Am Heart Assoc 5:2 10.1161/JAHA.116.004137 PMC5079053 27604456

[35] Schmidt M, Pedersen L, Sørensen HT (2014) The Danish civil registration system as a tool in epidemiology. Eur J Epidemiol 29:541–549 24965263 10.1007/s10654-014-9930-3

[36] Carstensen B, Kristensen JK, Marcussen MM, Borch-Johnsen K (2011) The national diabetes register. Scand J Public Health 39:58–61 21775353 10.1177/1403494811404278

[37] Adelborg K, Sundbøll J, Munch T, Frøslev T, Sørensen HT, Bøtker HE, Schmidt M (2016) Positive predictive value of cardiac examination, procedure and surgery codes in the Danish National Patient Registry: a population-based validation study. BMJ Open 6:e012817 27940630 10.1136/bmjopen-2016-012817 PMC5168662

[38] Sundbøll J, Adelborg K, Munch T, Frøslev T, Sørensen HT, Bøtker HE, Schmidt M (2016) Positive predictive value of cardiovascular diagnoses in the Danish National Patient Registry: a validation study. BMJ Open 6:e012832 27864249 10.1136/bmjopen-2016-012832 PMC5129042

