# Supplementary material for: Incidence of depression in patients with cardiovascular disease and type 2 diabetes: a nationwide cohort study
Source: Clin Res Cardiol. 2023 Oct 10;113(11):1523–33. doi: 10.1007/s00392-023-02311-3 (PMC11493809; doi:10.1007/s00392-023-02311-3)
Supplement: Supplementary file 1 — Supplementary file1 (DOCX 492 KB) [file 392_2023_2311_MOESM1_ESM.docx]

**Table S1:** Overview of the details regarding definition of study population, cardiovascular disease, comorbidities and medical treatment.

|  | | **Details** | **ICD-10 and ATC codes used** |
| --- | --- | --- | --- |
| **Study population** | | | |
| Type 2 diabetes | | Defined from treatment with glucose lowering drugs not in relation to type 1 diabetes, polycystic ovarian syndrome, gestational diabetes and use of liraglutide. | ATC: A10 |
| **Cardiovascular disease** | | | |
| Heart failure | | Defined from diagnosis codes including heart failure, cardiomyopathies, hypertensive heart failure. | ICD-10: I110, I130, I132, I42, I426-29, I50 |
| Stroke |  | | ICD-10: I60-I64, G45 |
| Myocardial infarction |  | | ICD-10: I21, I200 |
| Peripheral vascular disease | A patient had to receive one in-hospital contact diagnosis code of PAD or 2 consecutive out-hospital contact diagnosis codes of PAD to qualify for the diagnosis of PAD. We also included lower limp amputations | | ICD-10: 170, I739  KNHQ14, KNHQ17, KNFQ19 |
| **Outcome** | | | |
| Depression and anxiety | Diagnosis of depression/anxiety or prescription of an antidepressants/benzodiazepine. | | ICD-10: F320-F339, F34, F38, F39  F40-F43, F452  ATC: N06A, N05BA |
| **Comorbidity** | | | |
| Chronic kidney disease | Defined from diagnosis codes of chronic glomerulonephritis, chronic tubulointestinal nephropathy, chronic kidney disease, and diabetic and hypertensive nephropathy. | | ICD-10: E102, E112, E132, E 142, I120, M321B, M300,  M313, M319, N02-N08, N11-N12, N14, N158-N160,  N162-N164, N168, N18-N19, N26, Q612-Q613, Q615, Q619, |
| Atrial fibrillation |  | | ICD-10: I48 |
| Cancer | Defined from all cancer diagnosis codes, excluding non-melanoma skin cancer | | ICD-10: C00-C97 |
| Chronic obstructive pulmonary disease |  | | ICD-10: J42, J44 |
| Hypertension | Defined from combination  treatment with a least two classes of antihypertensive drugs  (Adrenergic α-antagonist, nonloop-  diuretics, vasodilators, betablockers,  calcium channel  blockers, and renin-angiotensin  system inhibitors) | | ATC: C02A, C02B, C02C, C02DA,  C02DB, C02DD, C02DG, C02L, C03A, C03B, C03D, C03E, C03X,  C07A, C07B, C07C, C07D, C07F,  C08, C09AA, C09BA, C09BB,  C09CA, C09DA, C09DB, C09XA02,  C09XA52 |
| Liver disease |  | |  |
| Gestational diabetes |  | | ICD-10: DO244B-E, BUBE02, D0244, DP700. |
| Any mental disease |  | | ICD-10: F00-F99  ICD-8: 290-299, 300-309 |
| **Concomitant pharmacotherapy** | | | |
| Statins | |  | ATC: C10A |
| Beta-blockers | |  | ATC: C07 |
| Mineralocorticoid receptor antagonists | |  | ATC: CO3D |
| ADP | |  | ATC: B01AC04, B01AC06, B01AC22, B01AC24, B01AC25, NO2BA01 |
| NOAC’s | |  | ATC: B01AF01, B01AF02, B01AE07 |
| Vitamin K antagonists | |  | ATC: B01AA03-04 |
| Ca antagonists | |  | ATC: C08 |
| Digoxin | |  | ATC: C01AA05 |
| Thiazide | |  | ATC CO3A |
| Renin angiotensin system inhibitors | | Including: angiotensin-converting-enzyme inhibitors, angiotensin-II receptor blockers | ATC: C09 |
| Loop diuretics | |  | ATC: C03CA01 |
| Insulin | |  | ATC: A10A |
| Metformin | |  | ATC: A10BA02 |
| Sulfonylurea | |  | ATC: A10BB |
| Thiazolidinedione | |  | ATC: A10BG |
| DDP-4 inhibitor | |  | ATC A10BH |
| GLP1-RA | |  | ATC: A10BJ |
| SGLT2 inhibitor | |  | ATC: A10BK |
| Combination of two glucose lowering agents. | |  | ATC: A10BD |
| Antipsychotic medication | |  | ATC :N05A |
| Lithium | |  | ATC: N05AN01 |

Abbreviations: ICD: International classification of disease, ATC: anatomical therapeutic code, ACE: angiotensin converting enzyme, ARB: angiotensin II receptor blocker, ADP: adenin diphosphate receptor, MRA mineralocorticoid receptor antagonists, DPP-4 dipeptidyl peptidase-4, GLP-1 glucagon-like peptide-1, SGLT2 sodium-glucose-cotransporter-2.

**Table S2: Incidence of depression stratified according to age**

| **Age** | **CVD group** | **Event** | **PY** | **Incidence rate per 1000 PY** | **Adjusted IRR** |
| --- | --- | --- | --- | --- | --- |
| <60y | MI | 2221 | 42408,9 | 52.4(39.2;68.6) |  |
| <60y | MI+T2D | 269 | 3225,4 | 83.4(66.5;103.3) | 1.36(1.2;1.54) |
| <60y | Stroke | 5454 | 34867,7 | 156.4(132.8;182.9) |  |
| <60y | Stroke+T2D | 436 | 1945,9 | 224.1(195.7;255.4) | 1.13(1.03;1.25) |
| <60y | PAD | 620 | 8201,8 | 75.6(59.5;94.7) |  |
| <60y | PAD+T2D | 204 | 1967 | 103.7(84.7;125.7) | 1.19(1.02;1.4) |
| <60y | HF | 888 | 13486,6 | 65.8(50.9;83.7) |  |
| <60y | HF+T2D | 142 | 1442,5 | 98.4(79.9;119.9) | 1.2(1;1.43) |
| <60y | => 2 CVD’s | 43 | 165,1 | 260.4(229.7;294) |  |
| <60y | => 2 CVD’s+T2D | 11 | 26,3 | 418.3(379.2;460.4) | 1.68(0.86;3.26) |
| 60-80y | MI | 4937 | 79327,4 | 62.2(47.7;79.7) |  |
| 60-80y | MI+T2D | 1096 | 10687,7 | 102.5(83.6;124.4) | 1.37(1.28;1.46) |
| 60-80y | Stroke | 15028 | 79425,9 | 189.2(163.2;218.2) |  |
| 60-80y | Stroke+T2D | 2469 | 10290,6 | 239.9(210.5;272.3) | 1.08(1.04;1.13) |
| 60-80y | PAD | 3237 | 29898,5 | 108.3(88.9;130.7) |  |
| 60-80y | PAD+T2D | 1349 | 9145,4 | 147.5(124.7;173.3) | 1.23(1.16;1.32) |
| 60-80y | HF | 4868 | 39428,2 | 123.5(102.7;147.3) |  |
| 60-80y | HF+T2D | 1641 | 9043 | 181.5(156.1;209.9) | 1.2(1.14;1.27) |
| 60-80y | => 2 CVD’s | 228 | 915,9 | 248.9(218.9;281.8) |  |
| 60-80y | => 2 CVD’s+T2D | 52 | 161,8 | 321.4(287.2;358.5) | 0.94(0.69;1.27) |
| >80y | MI | 4696 | 22665,3 | 207.2(179.9;237.4) |  |
| >80y | MI+T2D | 882 | 3221,1 | 273.8(242.3;308.2) | 1.16(1.08;1.25) |
| >80y | Stroke | 13237 | 37324 | 354.7(318.7;393.6) |  |
| >80y | Stroke+T2D | 1852 | 4316,2 | 429.1(389.5;471.7) | 1.12(1.06;1.17) |
| >80y | PAD | 2967 | 11521,1 | 257.5(227;290.9) |  |
| >80y | PAD+T2D | 844 | 2697,9 | 312.8(279.1;349.5) | 1.12(1.04;1.21) |
| >80y | HF | 10033 | 26356,6 | 380.7(343.4;420.9) |  |
| >80y | HF+T2D | 1963 | 4849,1 | 404.8(366.3;446.2) | 0.96(0.92;1.01) |
| >80y | => 2 CVD’s | 330 | 534,3 | 617.6(569.8;668.3) |  |
| >80y | => 2 CVD’s+T2D | 48 | 71,5 | 671.3(621.5;724.1) | 1.01(0.75;1.37) |

*The model was adjusted for gender, educational level, living situation, and comorbidities (atrial fibrillation, cancer, chronic obstructive pulmonary disease, hypertension, chronic kidney disease, and liver disease).

Abbreviations: y: year, T2D: type 2 diabetes, MI: myocardial infarction, PAD: peripheral artery disease, HF: heart failure, CVD: cardiovascular disease, PY: person-years, IRR: incidence rate ratio, CI: confidence interval, => 2 CVD’s: a combination of 2 or more cardiovascular disease subtypes.

**Table S3: Incidence of depression stratified according to age and gender**

| **Age** | **Sex** | **CVD group** | **Events** | **PY** | **IR per 1000 PY (95% CI)** | **Adjusted IRR* (95%CI)** |
| --- | --- | --- | --- | --- | --- | --- |
| <60y | Female | MI | 584 | 9615,8 | 60.7(46.4;78) |  |
| <60y | Female | MI+T2D | 74 | 627,2 | 118(97.7;141.3) | 1.28(1.1;1.49) |
| <60y | Female | Stroke | 2194 | 12756,4 | 172(147.3;199.7) |  |
| <60y | Female | Stroke+T2D | 115 | 532 | 216.2(188.3;247) | 1.21(1.07;1.36) |
| <60y | Female | PAD | 253 | 3332,6 | 75.9(59.8;95) |  |
| <60y | Female | PAD+T2D | 45 | 323,5 | 139.1(116.9;164.2) | 1.09(0.91;1.32) |
| <60y | Female | HF | 283 | 3633,9 | 77.9(61.6;97.2) |  |
| <60y | Female | HF+T2D | 34 | 299,1 | 113.7(93.8;136.6) | 1.25(1.01;1.53) |
| <60y | Female | => 2 CVD’s | 19 | 41,8 | 454.5(413.7;498.3) |  |
| <60y | Female | => 2 CVD’s+T2D | 2 | 1,4 | 1428.6(1355.5;1504.6) | 1.91(0.88;4.1) |
| <60y | Male | MI | 1637 | 32793,1 | 49.9(37;65.8) |  |
| <60y | Male | MI+T2D | 195 | 2598,2 | 75.1(59.1;94.1) | 1.61(1.27;2.06) |
| <60y | Male | Stroke | 3260 | 22111,3 | 147.4(124.6;173.2) |  |
| <60y | Male | Stroke+T2D | 321 | 1414 | 227(198.4;258.5) | 0.98(0.81;1.18) |
| <60y | Male | PAD | 367 | 4869,2 | 75.4(59.3;94.5) |  |
| <60y | Male | PAD+T2D | 159 | 1643,5 | 96.7(78.4;118) | 1.49(1.08;2.04) |
| <60y | Male | HF | 605 | 9852,8 | 61.4(47;78.8) |  |
| <60y | Male | HF+T2D | 108 | 1143,4 | 94.5(76.4;115.6) | 1.1(0.77;1.57) |
| <60y | Male | => 2 CVD’s | 24 | 123,3 | 194.6(168.2;223.9) |  |
| <60y | Male | => 2 CVD’s+T2D | 9 | 24,9 | 361.4(325.1;400.6) | 1.99(0.46;8.57) |
| 60-80y | Female | MI | 1669 | 22344,4 | 74.7(58.7;93.7) |  |
| 60-80y | Female | MI+T2D | 319 | 2666,4 | 119.6(99.1;143.1) | 1.41(1.3;1.52) |
| 60-80y | Female | Stroke | 6424 | 30255,2 | 212.3(184.7;242.9) |  |
| 60-80y | Female | Stroke+T2D | 864 | 3183,5 | 271.4(240.1;305.7) | 1.08(1.02;1.14) |
| 60-80y | Female | PAD | 1369 | 12001 | 114.1(94.1;137.1) |  |
| 60-80y | Female | PAD+T2D | 394 | 2319,7 | 169.8(145.2;197.3) | 1.18(1.09;1.28) |
| 60-80y | Female | HF | 1741 | 12596,2 | 138.2(116.1;163.3) |  |
| 60-80y | Female | HF+T2D | 534 | 2591 | 206.1(178.9;236.2) | 1.21(1.13;1.3) |
| 60-80y | Female | => 2 CVD’s | 96 | 230,4 | 416.7(377.6;458.7) |  |
| 60-80y | Female | => 2 CVD’s+T2D | 15 | 62,5 | 240(210.6;272.4) | 1.4(0.97;2.01) |
| 60-80y | Male | MI | 3268 | 56983 | 57.4(43.5;74.3) |  |
| 60-80y | Male | MI+T2D | 777 | 8021,3 | 96.9(78.6;118.2) | 1.29(1.14;1.46) |
| 60-80y | Male | Stroke | 8604 | 49170,7 | 175(150;202.9) |  |
| 60-80y | Male | Stroke+T2D | 1605 | 7107,1 | 225.8(197.3;257.3) | 1.09(1.01;1.17) |
| 60-80y | Male | PAD | 1868 | 17897,6 | 104.4(85.3;126.5) |  |
| 60-80y | Male | PAD+T2D | 955 | 6825,7 | 139.9(117.7;165.1) | 1.31(1.17;1.47) |
| 60-80y | Male | HF | 3127 | 26832 | 116.5(96.3;139.7) |  |
| 60-80y | Male | HF+T2D | 1107 | 6452 | 171.6(146.9;199.3) | 1.19(1.08;1.31) |
| 60-80y | Male | => 2 CVD’s | 132 | 685,5 | 192.6(166.4;221.8) |  |
| 60-80y | Male | => 2 CVD’s+T2D | 37 | 99,2 | 373(336.1;412.8) | 0.45(0.26;0.77) |
| >80y | Female | MI | 2206 | 9994,1 | 220.7(192.5;251.8) |  |
| >80y | Female | MI+T2D | 396 | 1467,2 | 269.9(238.7;304.1) | 1.26(1.14;1.39) |
| >80y | Female | Stroke | 7719 | 20344,4 | 379.4(342.2;419.6) |  |
| >80y | Female | Stroke+T2D | 940 | 2080,6 | 451.8(411.1;495.4) | 1.17(1.09;1.25) |
| >80y | Female | PAD | 1677 | 6364,2 | 263.5(232.6;297.3) |  |
| >80y | Female | PAD+T2D | 375 | 1168,2 | 321(286.8;358.1) | 1.15(1.03;1.27) |
| >80y | Female | HF | 5154 | 13475,7 | 382.5(345.1;422.8) |  |
| >80y | Female | HF+T2D | 927 | 2256,2 | 410.9(372.1;452.6) | 0.95(0.89;1.02) |
| >80y | Female | => 2 CVD’s | 179 | 263,2 | 680.1(629.9;733.2) |  |
| >80y | Female | => 2 CVD’s+T2D | 26 | 40,1 | 648.4(599.4;700.3) | 1.08(0.69;1.68) |
| >80y | Male | MI | 2490 | 12671,2 | 196.5(170;226) |  |
| >80y | Male | MI+T2D | 486 | 1753,9 | 277.1(245.4;311.7) | 1.07(0.96;1.19) |
| >80y | Male | Stroke | 5518 | 16979,6 | 325(290.6;362.3) |  |
| >80y | Male | Stroke+T2D | 912 | 2235,6 | 407.9(369.3;449.5) | 1.08(1.01;1.15) |
| >80y | Male | PAD | 1290 | 5156,9 | 250.2(220.2;283.2) |  |
| >80y | Male | PAD+T2D | 469 | 1529,8 | 306.6(273.2;342.9) | 1.11(0.99;1.24) |
| >80y | Male | HF | 4879 | 12880,9 | 378.8(341.6;418.9) |  |
| >80y | Male | HF+T2D | 1036 | 2592,9 | 399.6(361.4;440.8) | 0.97(0.91;1.04) |
| >80y | Male | => 2 CVD’s | 151 | 271,1 | 557(511.7;605.2) |  |
| >80y | Male | => 2 CVD’s+T2D | 22 | 31,4 | 700.6(649.7;754.5) | 0.95(0.63;1.43) |

*The model was adjusted for, educational level, living situation, and comorbidities (atrial fibrillation, cancer, chronic obstructive pulmonary disease, hypertension, chronic kidney disease, and liver disease). Abbreviations: y: year, T2D: type 2 diabetes, MI: myocardial infarction, PAD: peripheral artery disease, HF: heart failure, CVD: cardiovascular disease, PY: person-years, IRR: incidence rate ratio, CI: confidence interval, => 2 CVD’s: a combination of 2 or more cardiovascular disease subtypes.

**Table S4: Incidence of depression stratified according to duration of cardiovascular disease**

| **CVD duration** | **CVD group** | **Events** | **PY** | **IR per 1000 PY (95% CI)** | **Adjusted IRR (95% CI)** |
| --- | --- | --- | --- | --- | --- |
| 0-1y | MI | 6051 | 35977,5 | 168.2(143.7;195.6) |  |
| 0-1y | MI+T2D | 1144 | 4557,6 | 251(220.9;284) | 1.28(1.2;1.36) |
| 0-1y | Stroke | 23757 | 42357,9 | 560.9(515.4;609.3) |  |
| 0-1y | Stroke+T2D | 3217 | 4972,8 | 646.9(598;698.7) | 1.04(1;1.08) |
| 0-1y | PAD | 3082 | 13545,1 | 227.5(198.9;259.1) |  |
| 0-1y | PAD+T2D | 1050 | 3980,7 | 263.8(232.9;297.6) | 1.14(1.06;1.22) |
| 0-1y | HF | 8684 | 23007,2 | 377.4(340.3;417.5) |  |
| 0-1y | HF+T2D | 1955 | 4746,9 | 411.8(373;453.6) | 0.99(0.95;1.05) |
| 0-1y | => 2 CVD’s | 451 | 484 | 931.8(872.9;993.6) |  |
| 0-1y | => 2 CVD’s+T2D | 83 | 78,5 | 1057.3(994.5;1123) | 1.04(0.82;1.31) |
| >1y-3y | MI | 3369 | 61686,7 | 54.6(41.1;71.1) |  |
| >1y-3y | MI+T2D | 674 | 7361,2 | 91.6(73.8;112.4) | 1.33(1.22;1.44) |
| >1y-3y | Stroke | 6374 | 64742,8 | 98.5(80;120) |  |
| >1y-3y | Stroke+T2D | 967 | 7155,1 | 135.1(113.3;159.9) | 1.16(1.09;1.25) |
| >1y-3y | PAD | 2206 | 21370,7 | 103.2(84.3;125.1) |  |
| >1y-3y | PAD+T2D | 840 | 6041,7 | 139(116.9;164.1) | 1.27(1.17;1.38) |
| >1y-3y | HF | 4616 | 33975,4 | 135.9(114;160.8) |  |
| >1y-3y | HF+T2D | 1159 | 6648,3 | 174.3(149.4;202.2) | 1.09(1.02;1.17) |
| >1y-3y | => 2 CVD’s | 98 | 675,8 | 145(122.4;170.6) |  |
| >1y-3y | => 2 CVD’s+T2D | 20 | 112,9 | 177.1(152;205.2) | 1.04(0.64;1.68) |
| >3y-5y | MI | 2434 | 46737,4 | 52.1(38.9;68.3) |  |
| >3y-5y | MI+T2D | 429 | 5215,2 | 82.3(65.5;102.1) | 1.24(1.12;1.37) |
| >3y-5y | Stroke | 3588 | 44516,9 | 80.6(64;100.2) |  |
| >3y-5y | Stroke+T2D | 573 | 4425 | 129.5(108.2;153.8) | 1.34(1.23;1.46) |
| >3y-5y | PAD | 1536 | 14705,7 | 104.4(85.3;126.5) |  |
| >3y-5y | PAD+T2D | 507 | 3787,8 | 133.9(112.2;158.6) | 1.18(1.07;1.31) |
| >3y-5y | HF | 2489 | 22288,9 | 111.7(91.9;134.4) |  |
| >3y-5y | HF+T2D | 632 | 3939,4 | 160.4(136.5;187.2) | 1.2(1.09;1.31) |
| >3y-5y | => 2 CVD’s | 52 | 455,4 | 114.2(94.2;137.2) |  |
| >3y-5y | => 2 CVD’s+T2D | 8 | 68,2 | 117.3(97;140.5) | 0.86(0.41;1.81) |

*The model was adjusted for gender, age groups, educational level, living situation, and comorbidities (atrial fibrillation, cancer, chronic obstructive pulmonary disease, hypertension, chronic kidney disease, and liver disease).

Abbreviations: y: year, T2D: type 2 diabetes, MI: myocardial infarction, PAD: peripheral artery disease, HF: heart failure, CVD: cardiovascular disease, PY: person-years, IRR: incidence rate ratio, CI: confidence interval, => 2 CVD’s: a combination of 2 or more cardiovascular disease subtypes.

**Table S6: Baseline characteristics of patients diagnosed with depression**

|  | **MI (n=2738)** | **MI+T2D (n=397)** | **Stroke (n=10732)** | **Stroke+T2D (n=1345)** | **PAD (n=1584)** | **PAD+T2D (n=518)** | **HF (n=2162)** | **HF+T2D (n=463)** | **=>2CVD's (n=1686)** | **=>2CVD's+T2D (n=478)** |
| --- | --- | --- | --- | --- | --- | --- | --- | --- | --- | --- |
| Age at depression  median (IQR) | 70 (57, 80) | 73 (61, 80) | 73 (62, 81) | 75 (67, 81) | 75 (65.8, 82.0) | 73 (65, 81) | 82 (73, 88) | 79 (72, 85) | 77 (69, 85) | 75 (68, 83) |
| Female | 1,031 (37.7) | 140 (35.3) | 5,032 (46.9) | 503 (37.4) | 801 (50.6) | 183 (35.3) | 1,035 (47.9) | 182 (39.3) | 697 (41.3) | 164 (34.3) |
| Hypertension | 1676 (61.2) | 345 (86.9) | 6690 (62.3) | 1152 (85.7) | 929 (58.6) | 422 (81.5) | 1707 (79.0) | 429 (92.7) | 1341 (79.5) | 441 (92.3) |
| Atrial fibrillation | 449 (16.4) | 76 (19.1) | 2192 (20.4) | 334 (24.8) | 241 (15.2) | 96 (18.5) | 1207 (55.8) | 262 (56.6) | 677 (40.2) | 179 (37.4) |
| Cancer | 586 (21.4) | 82 (20.7) | 2,110 (19.7) | 310 (23.0) | 444 (28.0) | 122 (23.6) | 703 (32.5) | 151 (32.6) | 410 (24.3) | 123 (25.7) |
| COPD | 939 (34.3) | 155 (39.0) | 2762 (25.7) | 383 (28.5) | 621 (39.2) | 189 (36.5) | 1023 (47.3) | 235 (50.8) | 641 (38.0) | 196 (41.0) |
| CKD | 125 (4.6) | 71 (17.9) | 412 (3.8) | 176 (13.1) | 132 (8.3) | 112 (21.6) | 284 (13.1) | 134 (28.9) | 222 (13.2) | 155 (32.4) |

Abbreviations: IQR: interquartile range, T2D: type 2 diabetes, MI: myocardial infarction, PAD: peripheral artery disease, HF: heart failure, COPD: chronic obstructive pulmonary disease, CKD: chronic kidney disease, => 2 CVD’s: a combination of 2 or more cardiovascular disease subtypes.


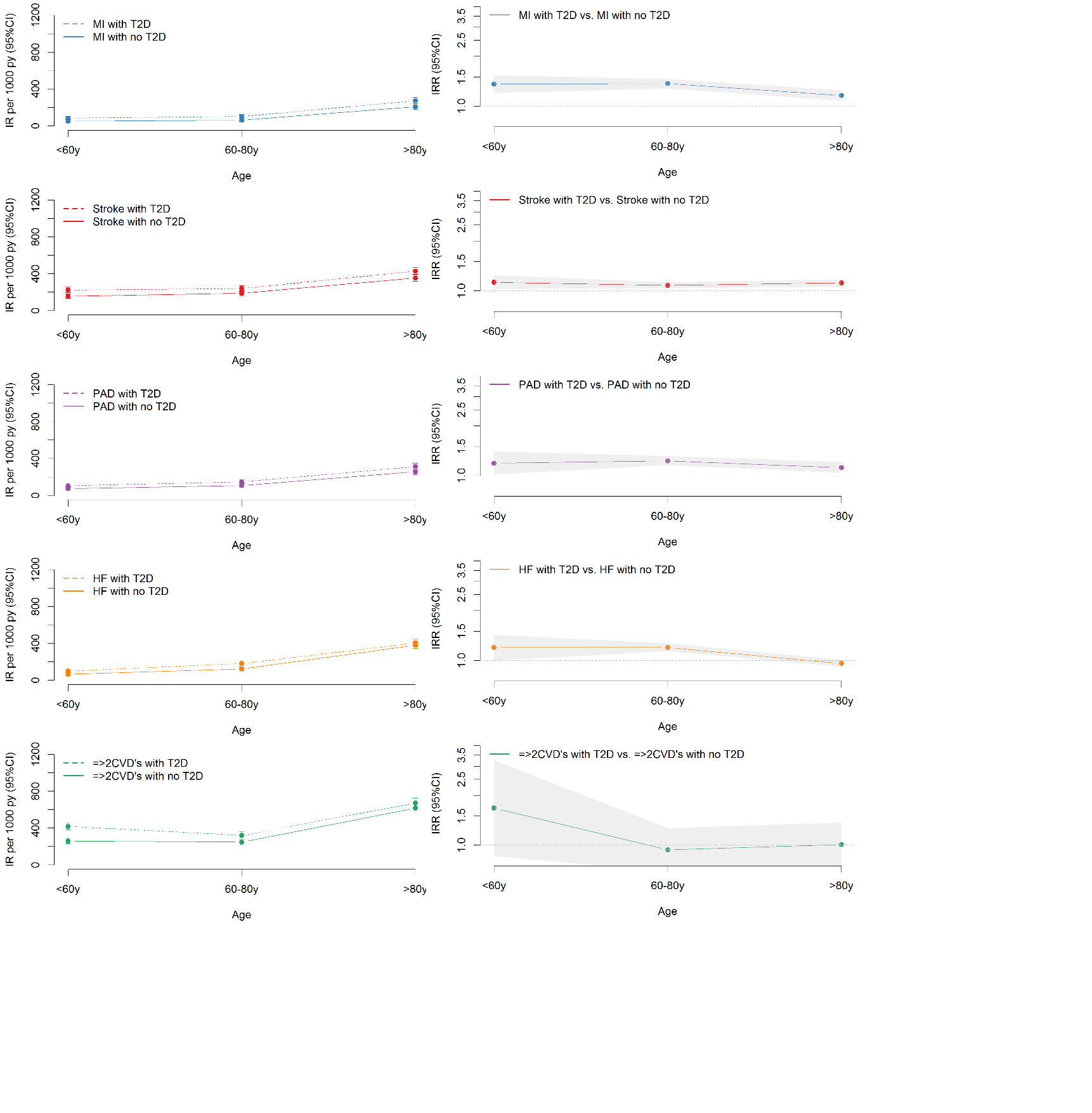


**Figure S1**: Crude incidence rates and adjusted incidence rate ratios of depression among subtypes of cardiovascular disease with type 2 diabetes compared to subtypes of cardiovascular disease free of type 2 diabetes according to age groups. The incidence rate ratios were adjusted for age, gender, educational level, living situation, and comorbidities (atrial fibrillation, cancer, chronic obstructive pulmonary disease, hypertension, chronic kidney disease and liver disease). Abbreviations: T2D: type 2 diabetes, CVD: cardiovascular disease, PY: person-years, IR: incidence rate per 1000 person years, CI: confidence interval, MI: myocardial infarction, HF: heart failure, PAD: peripheral artery disease.
